# Electroacupuncture Ameliorates Learning and Memory via Activation of the CREB Signaling Pathway in the Hippocampus to Attenuate Apoptosis after Cerebral Hypoperfusion

**DOI:** 10.1155/2013/156489

**Published:** 2013-10-20

**Authors:** Xiaohua Han, Xiuxiu Zhao, Min Lu, Fang Liu, Feng Guo, Jinghui Zhang, Xiaolin Huang

**Affiliations:** ^1^Department of Rehabilitation Medicine, Tongji Hospital, Tongji Medical College, Huazhong University of Science and Technology, Wuhan 430030, China; ^2^Department of Rehabilitation Medicine, the Third Affiliated Hospital, Sun Yat-Sen University, Guangzhou 510630, China

## Abstract

Studies have shown that electroacupuncture (EA) ameliorates learning and memory after ischemic injury. However, there have been few studies elucidating the mechanisms of EA on learning and memory in cerebral hypoperfusion. In this study, we explored the cAMP response element-binding protein (CREB) signaling pathway-mediated antiapoptotic action involved in EA-induced improvement of learning and memory. EA at GV20 and GV14 acupoints was applied in cerebral hypoperfusion rats. A Morris water maze task was performed, and the immunoreactivities of pCREB, Bcl-2, and Bax in the hippocampal CA1 area were evaluated by the Western blotting technique. Our findings indicated that (1) EA ameliorated spatial learning and memory impairment in cerebral hypoperfusion rats; (2) EA increased the immunoreactivities of pCREB and Bcl-2 and decreased the immunoreactivity of Bax; (3) intracerebroventricular administration of H89 (the inhibitor of protein kinase A) blocked EA-induced, pCREB-mediated antiapoptotic action and improved learning and memory. These results suggest that EA can ameliorate learning and memory via activation of the CREB signaling pathway in the hippocampus to attenuate apoptosis after cerebral hypoperfusion.

## 1. Introduction

Up to 64% of persons who have experienced a stroke have some degree of cognitive impairment [1], with up to a third developing frank dementia [2, 3]. Disorders of learning and memory, which are among the most common cognitive impairments, severely affect the curative efficacy and the quality of survivors' lives.

Some brain regions, such as the hippocampus, are more vulnerable to ischemic damage [4]. CA1 hippocampal pyramidal neurons exhibit cell death several days after ischemic injury [5]. Spatial memory in rats and humans is largely dependent on the hippocampus [6]. Hippocampal neuronal damage induced by ischemia, especially in the CA1 pyramidal neurons, is associated with spatial learning and memory impairment [7].

The cAMP response element-binding protein (CREB) is necessary for proliferation, growth, survival, and differentiation of all types of cells. In the brain, the CREB and CRE-mediated system is related to memory, learning, and synaptic transmission as well as in neuron survival. In neurons, CREB influences the expression of a variety of genes, of which brain-derived neurotrophic factor (BDNF) [8], c-fos [9], and Bcl-2 [10, 11] have been studied intensely. Because Bcl-2 is an antiapoptotic protein, CREB-mediated Bcl-2 expression is believed to be protective against ischemic insult [12]. Although CREB phosphorylation is increased in the whole hippocampus [13, 14], CREB activity is decreased in vulnerable CA1 neurons after ischemic insult [13]. Given that CREB activation is neuroprotective, an approach to induce CREB activation in vulnerable CA1 neurons might be a promising strategy to protect the brain against ischemic insult and ameliorate learning and memory.

Electroacupuncture (EA) delivers electrical stimulation to acupuncture points through acupuncture needles. EA has been recommended as a complementary therapy in the global scope of stroke rehabilitation. Several studies investigating the effectiveness of EA with cerebral ischemia have been conducted, and beneficial neuroprotective effects have been observed [15–18]. Both in animal models of cognitive impairment and in patients with deficiencies of learning and memory, some inspiring outcomes of EA have been reported [19, 20]. However, there have been few studies elucidating the mechanisms of EA on learning and memory in an animal model of cerebral hypoperfusion. Therefore, we determined whether EA could ameliorate learning and memory through the activation of the CREB signaling pathway in the hippocampus to attenuate apoptosis in a rat model of cerebral hypoperfusion.

## 2. Materials and Methods

### 2.1. Animals

Forty male Sprague-Dawley rats (Huazhong University of Science and Technology Experimental Animal Center, Wuhan, China) were used for the study. All animal experiments were approved by the ethics committee of the Tongji Medical College. All rats, each weighing 220 ± 20 g (age 7-8 weeks), were randomly divided into five groups (*n* = 8 in each group): a sham-operated control group (control group), a model group, an EA group, an EA combined with intracerebroventricular (ICV) injection of saline group (EA + NS group), and an EA combined with ICV injection of H89 group (EA + H89 group).

### 2.2. Surgical Procedures

Cerebral hypoperfusion was induced by permanent, bilateral common carotid artery occlusion (2-vessel occlusion, 2VO) in all groups except the control group. During the surgical procedures, rectal temperature was maintained at 37 ± 0.5°C with a heat lamp. The animals were anesthetized with 400 mg/kg chloral hydrate i.p. A ventral cervical incision was made in the midline to expose the common carotid arteries, which were gently separated from their sheaths and vagal nerves, and permanently ligated with surgical sutures. In the control group, the arteries were similarly exposed, but not ligated.

### 2.3. ICV Injection Protocol

The rats were anesthetized with chloral hydrate (400 mg/kg, i.p.), and their heads were fixed in a stereotaxic frame. H89 (2 *μ*g/*μ*L × 10 *μ*L, Beyotime Institute of Biotechnology, Shanghai, China.) was given intracerebroventricularly in the right hemisphere, 5–10 min before 2VO, similar to a previous study [21]. The coordinates for intracerebroventricular injections were as follows: 0.8 mm posterior to the bregma, 1.5 mm lateral to the midline, and 4.0 mm below the dural surface. The injection speed was controlled by a syringe pump at a rate of 2 *μ*L/min. The needle was retained in place for 5 min after injection. The EA combined with ICV injection of NS group underwent the same ICV injection protocol, but a corresponding volume of saline was injected instead of H89.

### 2.4. Treatment Protocol

**Figure 1:**
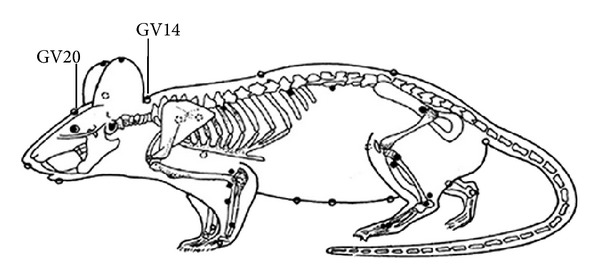
Rat schematic showing the location of the acupuncture points used in the study. GV14 stands for “Da zhui,” which is located on the posterior midline and in the depression below the spinous process of the seventh cervical vertebra; GV20 stands for “Bai hui,” which is located at the right midpoint of the parietal bone.

For EA treatment, “Bai hui” (GV20), which is located at the right midpoint of the parietal bone, and “Da zhui” (GV14), which is located on the posterior midline and in the depression below the spinous process of the seventh cervical vertebra (Figure 1), were located as in our previous study [22]. GV20 and GV14 were then electrically stimulated with a G6805-II electroacupuncture therapeutic apparatus (Shanghai Medical Electronic Apparatus Company, China) with continuous current at 20 Hz and 1-2 mA intensity for 20 minutes, once a day. EA was delivered to the rats from the day the 2VO model was established; the intervention lasted for 7 days. The rats in the control and model groups were fed in their cages without special intervention.

### 2.5. Morris Water Maze Task

On the 5th day after 2VO onset, the Morris water maze task was performed to test spatial learning and memory as described previously [23]. The maze consisted of a black circular pool (diameter 2 m, height 80 cm, and filled with water at 21-22°C to a height of 50 cm). The platform (10 cm in diameter) was hidden 2 cm below the surface of the water made opaque with ink. Several constant, large visual cues surrounded the tank at a height of 120–150 cm to facilitate orientation. The starting points were changed every day. Each trial lasted either until the rat found the platform or for 60 s. The rats rested on the platform for 20 s after each trial. The latency to find the submerged platform and the swimming distance were recorded. The speed of swimming was equal to the swimming distance divided by the latency. Two sessions of the four trials were conducted within a 4 h interval on the first testing day. The first session was considered a training procedure. One session out of the four trials was conducted daily during the next 2 testing days. Four hours after the last trial, the platform was removed for a 60 s spatial probe trial. The frequency of swimming across the platform in the training quadrant, that is, the previous location of the platform and the swimming distance in 60 s, was recorded (Ethovision; Noldus Information Technology, The Netherlands). The speed of swimming was equal to the swimming distance divided by 60 s.

### 2.6. Western Blotting Analysis

Total tissues of both sides of the hippocampal CA1 area were obtained, dissected into pieces, and extracted with the Total Protein Extraction Kit (ProMab, Richmond, CA, USA). A sample of each fraction was treated at 95°C for 10 min in SDS solubilization buffer and then separated on a 10% SDS-polyacrylamide gel (SDS-PAGE). The proteins were then transferred to nitrocellulose membranes (PIERCE, Rockford, IL, USA). Immunoblotting was performed using primary antibodies to pCREB (1 : 400, Santa Cruz, Inc., CA, USA), Bcl-2 (1 : 500, CST, Boston, MA, USA), and bax (1 : 1000, CST) for 2 h at room temperature and then incubated at 4°C overnight. After washing with PBS containing 0.05% Tween-20, the membranes were incubated with horseradish peroxidase-conjugated secondary antibodies (1 : 5000, Santa Cruz) for 1 h at room temperature. Protein bands were detected using an enhanced chemiluminescence method (ECL kit, Santa Cruz) according to the manufacturer's instructions. The optical densities of the specific bands of pCREB, Bcl-2, and bax were normalized based on the GAPDH band (1 : 500, Santa Cruz). Quantitation of bands was undertaken using Gel-Pro analyzer 4.0 software (Media Cybernetics, USA).

### 2.7. Statistical Analysis

The experimental results for each group are expressed as the mean ± SEM. A *t*-test (for the EA + NS and EA + H89 groups) and ANOVA with Student-Newman-Keuls post hoc analysis (for the control, model and EA groups) were performed. Statistically significant differences were set at *P* < 0.05.

## 3. Results

### 3.1. Morris Water Maze Task

**Figure 2:**
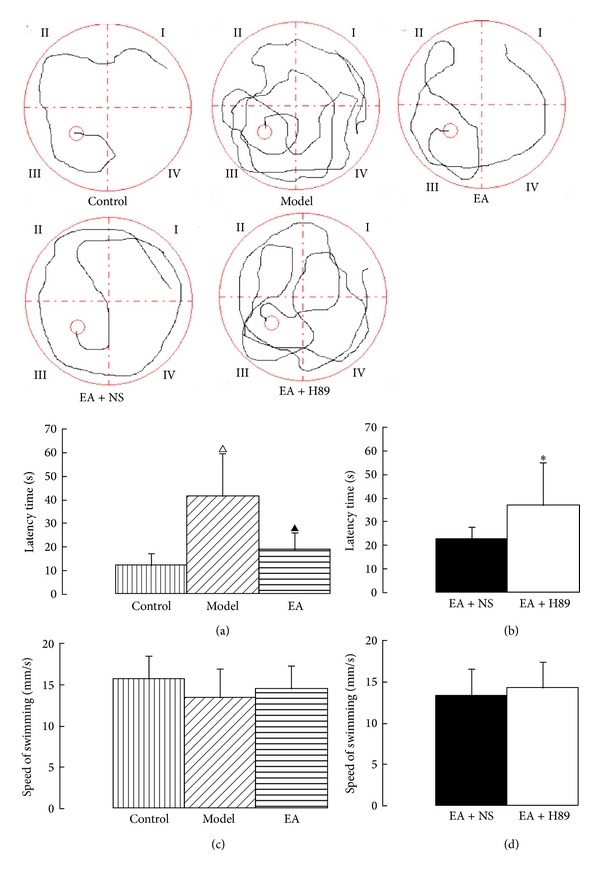
The traces of swimming in place navigation trials, the latency time to find the submerged platform, and the swimming speed of the five groups. The grey lines are the swimming traces, which end at the platforms (the small red circles in quadrant III). EA decreased the latency time of rats with cerebral hypoperfusion (a), and after ICV injection of H89, rats demonstrated prolonged latency (b), while there were no significant differences in the swimming speed either among the control, model, and EA groups or between the EA + NS and EA + H89 groups ((c) and (d)). The data are expressed as the mean ± SEM. ANOVA statistical analyses were performed to compare the means among the control, model, and EA groups; ^△^ *P* < 0.01 compared with the control group; ^▲^ *P* < 0.05 compared with the model group. A *t*-test was used to compare the means between the EA + NS and EA + H89 groups; *P < 0.05 compared with the EA + NS group.

**Figure 3:**
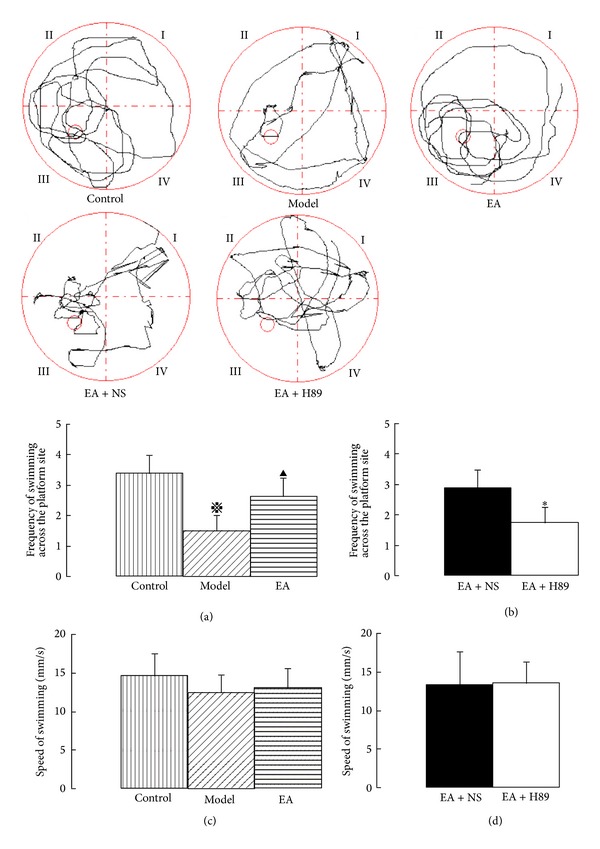
The traces of the 60 s spatial probe trials, the frequency of swimming across the platform site, and the swimming speed of the five groups. Cerebral hypoperfusion effected the spatial probe trial as demonstrated by the decreased frequency of swimming across the platform during the 60 s interval. In the EA group, the frequency increased (a); after ICV injection of H89, the increased frequency decreased (b), while there were no significant differences in the swimming speed either among the control, model, and EA groups or between the EA + NS and EA + H89 groups ((c) and (d)). The data are expressed as the mean ± SEM. ANOVA statistical analyses were performed to compare the means among the control, model, and EA groups;  ^**※**^ *P* < 0.05 compared with the control group; ^▲^ *P* < 0.05 compared with the model group. A *t*-test was used to compare the means between the EA + NS and EA + H89 groups; *P < 0.05 compared with the EA + NS group.

The rats were trained on the Morris water maze from the 5th day until the 7th day, followed by the place navigation trial and spatial probe trial. The latency to find the submerged platform, the swimming time across the platform, and the swimming distance were recorded by the same person who was blind to the grouping of the rats. In the place navigation trial, there were no significant differences in the swimming speed either among the control, model, and EA groups or between the EA + NS and EA + H89 groups (Figures 2(c)-2(d)), which was similar to in the spatial probe trial (Figures 3(c)-3(d)). The result indicated that the effect of 2VO was on learning and memory and not on motor performance. In the place navigation trial, cerebral hypoperfusion had an effect on water maze acquisition as demonstrated by the prolonged latency (41.40 ± 13.96 s) to locate a hidden platform in the model group rats compared with the control group (12.28 ± 4.53 s, *P* < 0.01). In the EA group (18.48 ± 5.74 s), the latency was shorter than that of the model group (*P* < 0.05; Figure 2(a)). After ICV injection of H89, rats demonstrated prolonged latency (36.95 ± 11.94 s) compared with the EA+NS group (22.67 ± 5.53 s, *P* < 0.05; Figure 2(b)). In the spatial probe trial, the frequency of swimming across the platform in 60 s in the model group (1.50 ± 0.53) decreased compared with that of the control group (3.38 ± 0.93, *P* < 0.05). In the EA group (2.63 ± 0.53), the frequency increased compared with that of the model group (*P* < 0.05; Figure 3(a)). However, in the EA + H89 group (1.75 ± 0.73), the frequency decreased compared with that of the EA + NS group (2.88 ± 0.64, *P* < 0.05; Figure 3(b)).

### 3.2. Effects of EA on the Immunoreactivity of pCREB

**Figure 4:**
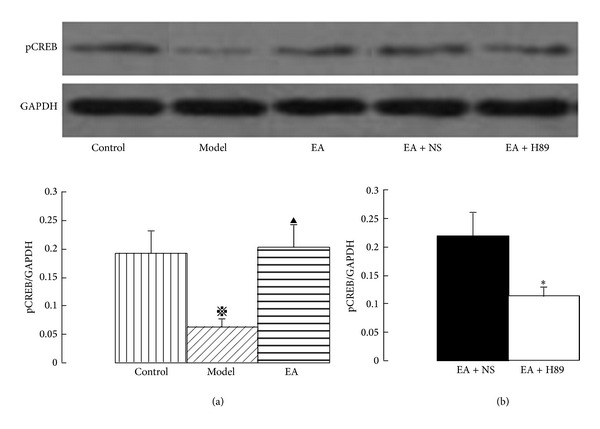
Western blotting showing immunoreactivity of phosphorylated CREB in the hippocampal CA1 area. The pCREB protein values were calculated as a ratio of pCREB protein to GAPDH. EA increased the expression of pCREB protein (a), and the EA-induced activation of pCREB was markedly inhibited by ICV injection of H89 (b). The data are expressed as the mean ± SEM. ANOVA statistical analyses were performed to compare the means among the control, model, and EA groups;  ^**※**^ *P* < 0.05 compared with the control group; ^▲^ *P* < 0.05 compared with the model group. A *t*-test was used to compare the means between the EA + NS and EA + H89 groups; *P < 0.05 compared with the EA + NS group.

The effects of EA on the immunoreactivity of pCREB in the hippocampal CA1 area were examined by Western blotting analysis (Figure 4). A significant decrease in the immunoreactivity of pCREB was observed in the hippocampal CA1 area after 2VO. Treatment with EA significantly attenuated the decrease in the immunoreactivity of pCREB (Figure 4(a)). Likewise, the immunoreactivity of pCREB increased in the EA + NS group, but this increase was markedly inhibited by ICV injection of H89 (Figure 4(b)).

### 3.3. Effects of EA on the Immunoreactivities of Bcl-2 and Bax

**Figure 5:**
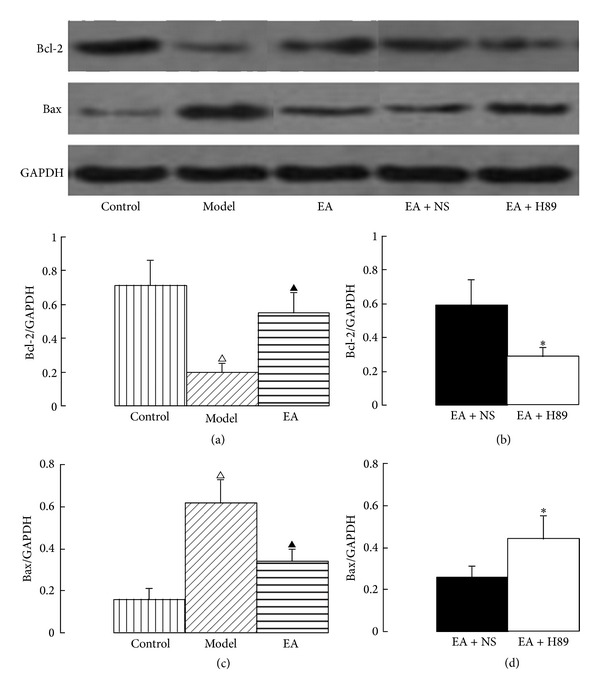
Western blotting showing immunoreactivity of apoptosis-related proteins in the hippocampal CA1 area. The Bcl-2 and Bax protein values were calculated as a ratio of Bcl-2 and Bax protein to GAPDH. EA increased the expression of Bcl-2 and decreased the expression of Bax ((a) and (c)). However, the antiapoptotic action of EA was markedly inhibited by ICV injection of H89 ((b) and (d)). The data are expressed as the mean ± SEM. ANOVA statistical analyses were performed to compare the means among the control, model, and EA groups; ^△^ *P* < 0.01 compared with the control group; ^▲^ *P* < 0.05 compared with the model group. A *t*-test was used to compare the means between the EA + NS and EA + H89 groups; *P < 0.05 compared with the EA + NS group.

One of the main mechanisms involved in the induction of the apoptotic pathway is the decrease in Bcl-2 levels or, alternatively, an increase in Bax levels. The Bcl-2 family is involved in cell death processes and plays a pivotal role in the cellular apoptotic machinery [24]. Bcl-2 is an antiapoptotic protein, and Bax exhibits proapoptotic activity [24, 25]. To investigate whether EA could attenuate apoptosis in the hippocampus after cerebral hypoperfusion, the immunoreactivities of Bcl-2 and Bax in the hippocampal CA1 area were examined by Western blotting analysis. Cerebral hypoperfusion had an effect on apoptosis in the hippocampal CA1 area as demonstrated by the decreased immunoreactivity of Bcl-2 and increased immunoreactivity of Bax in the model group rats compared with that of the control group. In the EA group, the immunoreactivity of Bcl-2 significantly increased and the immunoreactivity of Bax significantly decreased compared with that of the model group (Figures 5(a) and 5(c)). However, in EA + H89 group, the immunoreactivity of Bcl-2 significantly decreased and the immunoreactivity of Bax significantly increased compared with that of the EA + NS group (Figures 5(b) and 5(d)).

## 4. Discussion

In the present study, we provide evidence that EA ameliorates learning and memory through the activation of the CREB signaling pathway in the hippocampal CA1 area to attenuate apoptosis after cerebral hypoperfusion. The ultimate goal of acupuncture is to restore internal balance and harmony. As one form of acupuncture, EA delivers electrical stimulation to the acupoints through needles. A considerable number of investigations have been conducted studying the effectiveness of EA in treating cerebral ischemia. Several beneficial outcomes have been observed, including protection of neurological function by decreasing the infarct volume and activation of the retinoic acid signaling pathway [26], improved cerebral blood flow and attenuation of moderate ischemic injury via an endothelial mechanism [27], and activation of the cerebral structures related to motor function on the bilateral hemispheres [16]. In the present study, the model group showed prolonged latency to locate a hidden platform and decreased frequency of swimming across the platform during a 60 s period compared with the control group. After EA treatment, the latency was shortened and the frequency increased in the EA group. These results suggest that EA could ameliorate learning and memory after cerebral ischemia, consistent with previous reports [18, 28].

EA is a proven, effective therapy for ameliorating learning and memory, but the underlying mechanisms remain uncertain. In this study, we investigated the immunoreactivity of pCREB and apoptotic status in the hippocampal CA1 area. Though there were some measurements of apoptosis (i.e., TUNEL labeling, activated Caspase 3, etc.) to document an apoptotic mechanism, we explored the CREB signaling pathway-mediated antiapoptotic action in our study. The expression of Bcl-2 is mediated by CREB [10–12], and pCREB plays a role in the decrease of Bax expression [29]. Moreover, Bcl-2 is an antiapoptotic protein and Bax exhibits proapoptotic activity. So, a special emphasis was put on Bcl-2 and Bax to investigate the apoptotic status in our study. The model group showed decreased immunoreactivity of pCREB in the hippocampal CA1 area after 2VO, consistent with Walton's report [13], decreased immunoreactivity of Bcl-2, and increased immunoreactivity of Bax. After treatment, increased immunoreactivity of pCREB and Bcl-2 and decreased immunoreactivity of Bax were observed in the EA group. These results suggest that EA is involved in proapoptotic activity, which may be mediated via activation of CREB phosphorylation.

Furthermore, the importance of activating the CREB pathway after EA was demonstrated by ICV injection of H89. Nuclear CREB is phosphorylated and activated by cAMP-dependent protein kinase (PKA) and, consequently, binds to the cAMP response element (CRE) of target genes [30]. As the inhibitor of protein kinase A, H89 can inhibit CREB phosphorylation [10]. To control against nonspecific effects, a control ICV injection of NS was used. In the present study, ICV injection of H89 inhibited the improvement of spatial learning and memory after EA treatment. Moreover, ICV injection of H89 significantly inhibited CREB phosphorylation, reduced the expression of antiapoptotic Bcl-2, and significantly increased the expression of proapoptotic Bax. These results suggest that EA treatment could improve learning and memory after cerebral hypoperfusion via the CREB-mediated signaling pathway and subsequent activation of target proteins, such as the antiapoptotic protein Bcl-2, to attenuate apoptosis.

## 5. Conclusions

In conclusion, the present study demonstrated that EA can improve cognitive impairment after cerebral hypoperfusion. The protective effect of EA was associated with its antiapoptotic mechanism through the pCREB-mediated signaling pathway. These results indicate that EA could be a promising candidate for complementary therapy to ameliorate learning and memory after cerebral ischemia.
